# A mechanistic basis for potent, glycoprotein B-directed gammaherpesvirus neutralization

**DOI:** 10.1099/vir.0.032177-0

**Published:** 2011-09

**Authors:** Daniel L. Glauser, Anne-Sophie Kratz, Laurent Gillet, Philip G. Stevenson

**Affiliations:** 1 Division of Virology, Department of Pathology, University of Cambridge, Cambridge, UK; 2 Immunology-Vaccinology, Faculty of Veterinary Medicine, University of Liège, Liège, Belgium

## Abstract

Glycoprotein B (gB) is a conserved, essential component of gammaherpes virions and so potentially vulnerable to neutralization. However, few good gB-specific neutralizing antibodies have been identified. Here, we show that murid herpesvirus 4 is strongly neutralized by mAbs that recognize an epitope close to one of the gB fusion loops. Antibody binding did not stop gB interacting with its cellular ligands or initiating its fusion-associated conformation change, but did stop gB resolving stably to its post-fusion form, and so blocked membrane fusion to leave virions stranded in late endosomes. The conservation of gB makes this mechanism a possible general route to gammaherpesvirus neutralization.

## Introduction

Vaccination provides a cornerstone of antiviral intervention. However, herpesviruses routinely transmit from immunocompetent hosts (Klein, 1989; Gorman *et al.*, 2006), and while vaccination can dampen down their acute infections it has not prevented their persistence or transmission (Baigent *et al.*, 2006). Neutralizing antibodies are a key component of antiviral immunity (Zinkernagel & Hengartner, 2006), so limited infection control by natural and vaccine-primed immune responses suggests that *in vivo* herpesvirus neutralization is not easily achieved.

Murid herpesvirus 4 (MuHV-4), a close relative of the Kaposi’s sarcoma-associated herpesvirus (Efstathiou *et al.*, 1990; Virgin *et al.*, 1997), provides a way to analyse how gammaherpesviruses and antibodies interact. Immune sera block MuHV-4 binding to fibroblasts (Gill *et al.*, 2006), but promote myeloid cell infection via IgG Fc receptor binding (Rosa *et al.*, 2007; Smith *et al.*, 2007) and poorly block host entry (Gillet *et al.*, 2007a). Therefore, good *in vivo* neutralization may have to target processes downstream of binding such as membrane fusion.

Fusion requires the conserved virion glycoproteins B (gB) and H (gH) (Spear & Longnecker, 2003; Hutt-Fletcher, 2007). A fusogenic role for gB is supported by structural homology between herpesvirus gBs (Heldwein *et al.*, 2006; Backovic *et al.*, 2009) and the post-fusion vesicular stomatitis virus glycoprotein G (VSV-G) (Roche *et al.*, 2006). The MuHV-4 gB undergoes a marked antigenic change when virion capsids are released from late endosomes: some gB-specific mAbs (archetype BN-1A7) recognize only extracellular virions; others (archetype MG-1A12) recognize only those in late endosomes; relatively few recognize both (Gillet *et al.*, 2008a). The gB of extracellular virions must be pre-fusion. At least some of that in late endosomes must be post-fusion, and the uniformity of the virion switch from BN-1A7^+^MG-1A12^−^ to BN-1A7^−^MG-1A12^+^ in late endosomes implies that all gB becomes post-fusion. Treating cells with concanamycin A to raise the endosomal pH blocks both the gB conformation switch and capsid release. However, low pH alone triggers neither (Gillet *et al.*, 2008a). Thus, the fusion mechanism seems to involve pH but is not simply pH driven.

gB-directed neutralization has been explored extensively for human cytomegalovirus (HCMV) (Cranage *et al.*, 1986; Ohlin *et al.*, 1993; Speckner *et al.*, 1999). For both it and herpes simplex virus (HSV), gB-specific neutralizing mAbs can block virus penetration (Highlander *et al.*, 1988; Navarro *et al.*, 1993). Such neutralization is much less readily achieved for MuHV-4 (Gillet *et al.*, 2006). However, HSV and HCMV neutralization is generally defined as 50 % plaque reduction. The less uniformly lytic replication of MuHV-4 (May *et al.*, 2004) makes such minor reductions hard to reproduce; such reductions can also be achieved by virion cross-linking. Therefore, our more stringent (although equally arbitrary) cut-off for significant MuHV-4 neutralization is 80 % plaque reduction. It cannot be assumed that 50 % neutralization will become 80 % with more antibody. For example, *O*-glycosylation limits neutralization directed against the MuHV-4 gB N terminus regardless of antibody dose (Gillet & Stevenson, 2007a). Thus, weak neutralization may be noted more often for HCMV and HSV than for MuHV-4.

Another puzzle with HSV and HCMV is that many neutralization epitopes map to prominent features of post-fusion gB (Heldwein *et al.*, 2006). Because HSV and HCMV fuse *in vitro* with the plasma membrane (Spear & Longnecker, 2003), some post-fusion gB epitopes might become accessible to extracellular antibody before actual capsid release. The endocytic infection of MuHV-4 (Gill *et al.*, 2006) by contrast segregates fusion from free antibody, and mAbs (*n*>30) specific for post-fusion gB – that is those recognizing virion gB only after capsid release – do not neutralize (our unpublished data). Thus, endocytic infection may increase the difficulty of gB-directed neutralization.

Where gB-directed MuHV-4 neutralization does occur, the gB N terminus is a frequent target (Gillet *et al.*, 2006). This is consistent with results from other herpesviruses (Ohlin *et al.*, 1993; Akula *et al.*, 2002; Okazaki *et al.*, 2006). The MuHV-4 gB N terminus is redundant for infectivity, so antibodies binding here must neutralize by steric hindrance and have been effective only as pentameric IgMs (Gillet & Stevenson, 2007a). Several other MuHV-4 gB neutralization epitopes show the same dependence on high antibody avidity (Gillet *et al.*, 2008a). Such neutralization has limited relevance to vaccination, where most antibodies are IgG. However, we have recently identified two potently neutralizing MuHV-4 gB-specific IgGs. While immunization with recombinant gB boosted neutralization in only a minority of carrier mice and did not elicit neutralizing antibodies in naive mice (May & Stevenson, 2010), a more refined immunogen that selectively presents key gB epitopes might be more effective. In order to develop such an approach, we analysed here how IgG-mediated gB-directed neutralization works.

## Results

### Mapping a potent gB-specific neutralization epitope

**Fig. 1.**
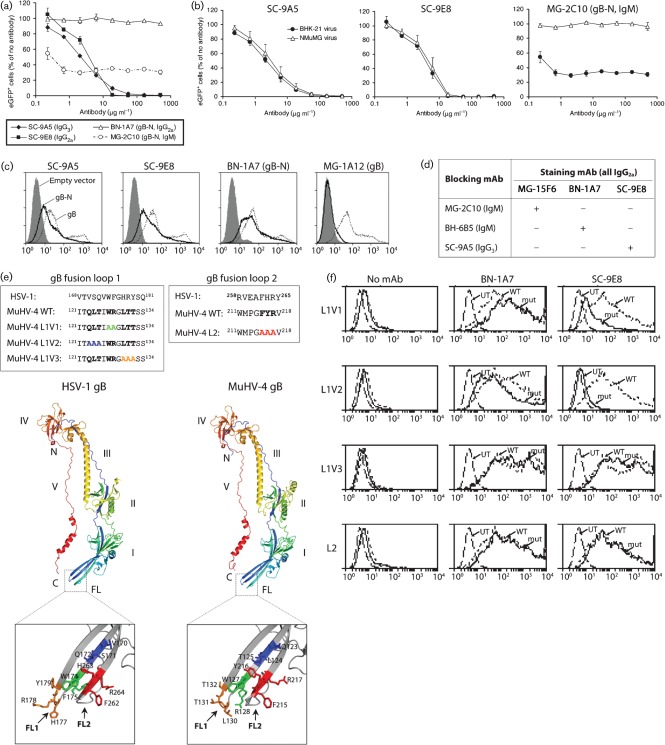
(a) Virus neutralization by gB-specific mAbs SC-9A5 and SC-9E8. Bacterial artificial chromosome (BAC)^+^ MuHV-4 (0.1 p.f.u. per cell) was incubated with gB-specific mAbs SC-9A5 (IgG_3_), SC-9E8 (IgG_2a_), BN-1A7 (IgG_2a_, non-neutralizing) or MG-2C10 (IgM, neutralizing) before being added to BHK-21 cells. After overnight incubation (37 °C) in the presence of 100 µg phosphonoacetic acid ml^−1^ to prevent further virus spread, eGFP^+^ cells were enumerated by flow cytometry and are shown relative to untreated virus. Each point shows the mean±sem of two experiments. By chi-squared test comparing the proportions of eGFP^+^ and eGFP^−^ cells, mAbs SC-9A5 and SC-9E8 gave significantly less neutralization than mAb MG-2C10 at <10 µg ml^−1^ (lower ID_50_) but at >10 µg ml^−1^ neutralization was significantly more complete (*P*<10^−5^). (b) SC-9A5 and SC-9E8 neutralize both fibroblast and epithelial cell-derived virions. BAC^+^ MuHV-4 (0.1 p.f.u. per cell) grown in either BHK-21 fibroblasts or NMuMG epithelial cells was incubated with antibody then used to infect BHK-21 cells as in (a). Despite a low ID_50_, MG-2C10 fails to neutralize BHK-21 cell-derived virions completely and NMuMG cell-derived virions at all because its epitope is variably masked by *O*-linked glycans. Each point shows the mean±sem of two experiments. (c) The SC-9A5/SC-9E8 epitope is located in the N-terminal half of gB. 293T cells were transfected with glycosyl-phosphatidyl-inositol (GPI)-linked gB fragments comprising the entire extracellular domain (gB), its N-terminal 423 aa residues (gB-N) or empty vector (grey histogram), then stained with gB-specific mAbs and analysed by flow cytometry. BN-1A7 recognizes an epitope in gB-N; MG-1A12 recognition requires the full-length gB extracellular domain. (d) SC-9A5 and SC-9E8 recognize identical or overlapping epitopes distinct from those recognized by neutralizing IgMs MG-2C10 and BH-6B5. MuHV-4-infected BHK-21 cells (2 p.f.u. per cell, 18 h) were incubated with the indicated blocking mAbs, and then the indicated IgG_2a_ staining mAbs followed by an IgG_2a_-specific, fluorescently labelled secondary antibody. The cells were then analysed for fluorescence by flow cytometry. ‘+’ = Reduced binding of the staining mAbs; ‘−’ = no effect. (e) Mutagenesis of the putative MuHV-4 gB fusion loops. Fusion loops 1 and 2 of the HSV gB and the homologous regions of the MuHV-4 gB are shown, together with three mutations we introduced into loop 1 (L1V1, L1V2 and L1V3) and one mutation we introduced into loop 2 (L2). The cartoons show the published structure of the HSV-1 gB ectodomain (Heldwein *et al.*, 2006) and the predicted structure of the corresponding amino acid residues 60–680 of the MuHV-4 gB ectodomain. The positions of the mutated MuHV-4 gB residues and the homologous HSV-1 gB residues are shown in green for L1V1, in blue for L1V2, in orange for L1V3 and in red for L2. (f) SC-9A5 and SC-9E8 bind close to gB fusion loop 1. 293T cells were transfected with GPI-linked wild-type (WT) gB, one of the mutants (L1V1, L1V2, L1V3 or L2) or left untransfected (UT), then stained with gB-specific mAbs and analysed by flow cytometry. L1V3 was introduced into full-length gB. L1V1, L1V2 and L2 expressed poorly in this form and so were introduced into gB-N. Each was matched with the appropriate WT (gB or gB-N). mAbs SC-9A5 and SC-9E8 recognized L1V1 and L1V2 significantly less well than WT, L1V3 or L2 (*P*<10^−5^ by *t*-test). Equivalent data were obtained in two further experiments.

A large-scale screen of B-cell hybridomas from MuHV-4 carrier mice identified SC-9A5 (IgG_3_) and SC-9E8 (IgG_2a_) as potent neutralizing mAbs (Fig. 1a). SC-9A5 was consistently more effective at low dose, whereas SC-9E8 was more effective at high dose, possibly reflecting an influence of isotype on mAb binding (Greenspan & Cooper, 1995). Unlike mAb MG-2C10 which is blocked from recognizing normal murine mammary gland (NMuMG) cell-derived virions by *O*-linked glycans (Gillet & Stevenson, 2007a), SC-9A5 and SC-9E8 neutralized both NMuMG and baby hamster kidney (BHK-21) cell-derived virions (Fig. 1b). Note that while MG-2C10 has a lower ID_50_, SC-9A5/SC-9E8 show much better maximal neutralization.

Like all our mAbs that recognize extracellular virion gB, SC-9A5 and SC-9E8 recognized the gB N-terminal half (gB-N) (Fig. 1c). Blocking experiments (Fig. 1d) established that the SC-9E8 epitope was distinct from that of MG-2C10 (Gillet *et al.*, 2006) or another neutralizing IgM, BH-6B5 (Gillet *et al.*, 2008a), but overlapped that of SC-9A5. The N-terminal gB domains include its putative fusion loops (Heldwein *et al.*, 2006; Backovic *et al.*, 2007; Hannah *et al.*, 2009), which are analogous to the fusion loops of VSV-G (Roche *et al.*, 2007). Fig. 1(e) compares the HSV-1 gB structure (Heldwein *et al.*, 2006) with that predicted for MuHV-4. Residues identified as critical for HSV fusion (Hannah *et al.*, 2009) are shown, together with analogous mutations we made in the MuHV-4 loops (L1V1, L1V2, L1V3 and L2). Fig. 1(f) shows how these mutations affected gB recognition by SC-9E8 and a control mAb, BN-1A7. Mutating fusion loop 2 had no effect. Mutations L1V1 and L1V2 around loop 1 substantially reduced recognition by SC-9E8 without affecting BN-1A7. A more precise loop 1 mutation (L1V3) affected neither. Therefore, the SC-9E8 epitope was not fusion loop 1 itself – not surprisingly because the fusion loops should not be accessible on extracellular virions – but appeared to be close to loop 1. It could involve the L1V1 and L1V2 mutation sites directly, or be affected by local conformation changes caused by the mutations.

### The SC-9E8/SC-9A5 epitope is exclusive to pre-fusion gB

**Fig. 2.**
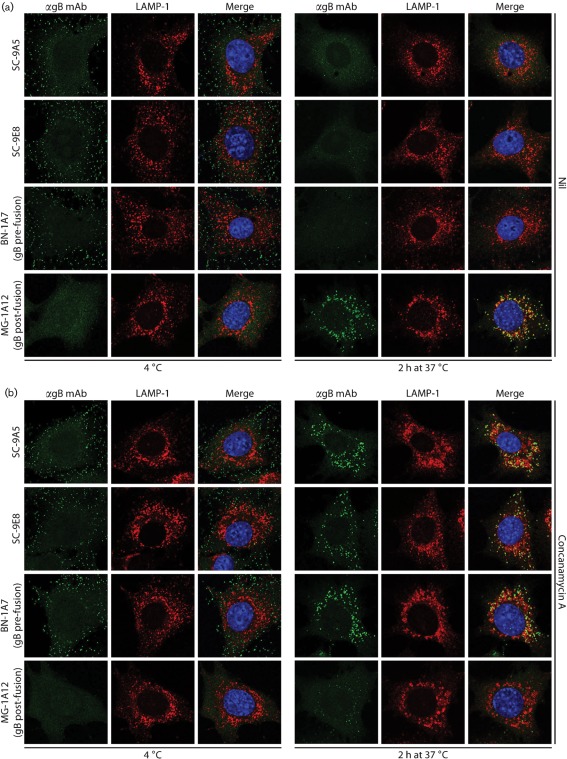
SC-9A5 and SC-9E8 recognize pre-fusion gB. (a) NMuMG cells were incubated with MuHV-4 (3 p.f.u. per cell, 2 h, 4 °C), washed and then either fixed immediately or first further incubated (2 h, 37 °C) to allow virion endocytosis. The cells were then stained with mAbs SC-9A5, SC-9E8, BN-1A7 or MG-1A12 (green). BN-1A7 recognizes only pre-fusion gB; MG-1A12 recognizes only post-fusion gB. The cells were also stained for LAMP-1 (red) and counter-stained with DAPI (blue). Equivalent data were obtained in two further experiments. In this and all subsequent figures, the data shown are representative of at least 100 cells examined. Note that MuHV-4 plaque titres underestimate virion numbers 10–100-fold. (b) Infections and stainings were performed as in (a), except that the cells were incubated with 1 µM concanamycin A before adding virus (2 h, 37 °C), as well as during virus binding and endocytosis, so as to raise the endosomal pH and prevent virion membrane fusion. Equivalent data were obtained in two further experiments.

That SC-9E8 and SC-9A5 recognize pre-fusion gB was confirmed by staining virions bound to cells at 4 °C (Fig. 2a). Here gB must be pre-fusion, since MuHV-4 capsids are released only after endocytosis. After a further 2 h incubation at 37 °C, during which virions reach lysosomal-associated membrane protein (LAMP-1)^+^ late endosomes and fuse, recognition by SC-9E8 and SC-9A5 was lost. This pattern conformed to recognition by the pre-fusion gB-specific mAb BN-1A7, and was opposite to that by the post-fusion gB-specific mAb MG-1A12. When membrane fusion was blocked by concanamycin A, gB recognition by BN-1A7 was preserved and that by MG-1A12 was prevented, indicating that gB remained in its pre-fusion form. Recognition by SC-9E8 and SC-9A5 was also preserved (Fig. 2b). Therefore, the SC-9E8/SC-9A5 epitope, like that of BN-1A7, was specific to pre-fusion gB.

### Neutralized virions bind to cells but cannot fuse

**Fig. 3.**
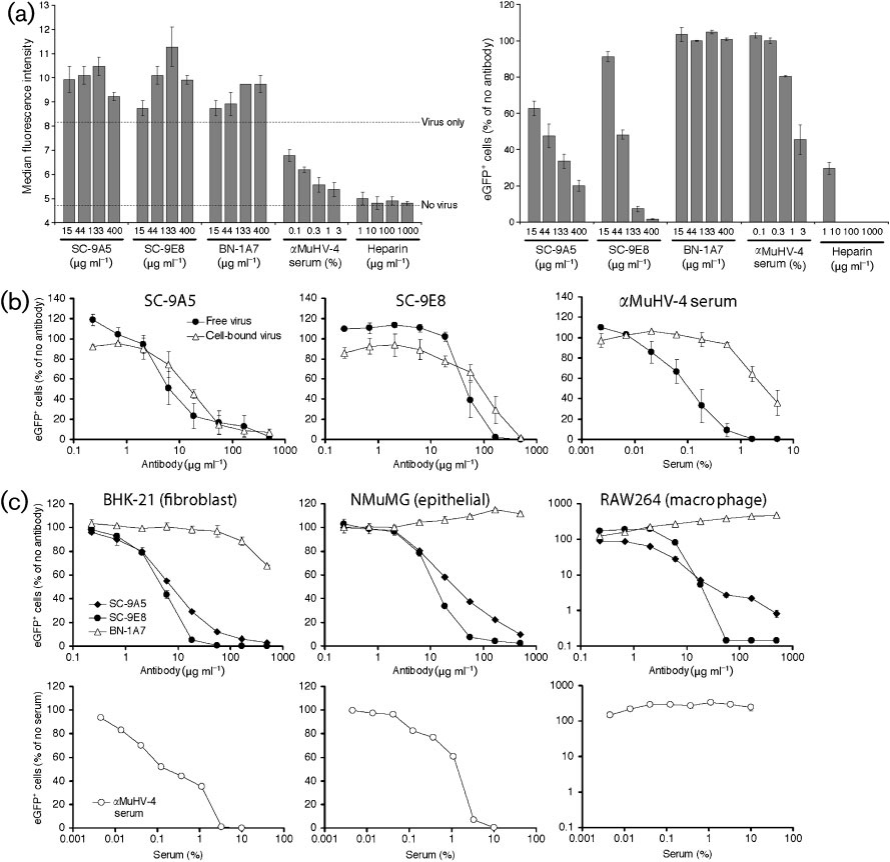
(a) SC-9A5 and SC-9E8 inhibit infection without reducing cell binding. To assess virus binding (left panel), gM-eGFP^+^ virions (3 p.f.u. per cell) were pre-incubated with mAbs, MuHV-4-immune serum or heparin (2 h, 37 °C) before binding to NMuMG cells (2 h, 4 °C). Unbound virions were removed by washing and the cells analysed immediately for eGFP fluorescence by flow cytometry. To assess virus entry (right panel), BAC^+^ MuHV-4 (0.1 p.f.u. per cell) was pre-incubated as above then added to NMuMG cells, which were analysed for viral eGFP expression after overnight incubation (37 °C) with 100 µg phosphonoacetic acid ml^−1^. The bars show mean±sem values from two independent experiments. (b) SC-9A5 and SC-9E8 neutralize both cell-bound and cell-free virions. To neutralize cell-free virions, BAC^+^ MuHV-4 (0.3 p.f.u. per cell) was pre-incubated with mAbs or immune serum (2 h, 4 °C), then bound to BHK-21 cells (2 h, 4 °C). Unbound virions were removed by washing with PBS. To neutralize cell-bound virus, BHK-21 cells were incubated with BAC^+^ MuHV-4 (0.3 p.f.u. per cell, 2 h, 4 °C), washed, incubated with mAbs or immune serum (2 h, 4 °C), then washed again. All cells were then incubated overnight (37 °C) with 100 µg phosphonoacetic acid ml^−1^ and analysed for viral eGFP expression by flow cytometry. For SC-9A5 and SC-9E8 ID_50_ values for either method differed <threefold; for immune serum the difference was >30-fold. (c) SC-9A5 and SC-9E8 neutralize MuHV-4 for fibroblast, epithelial cell and macrophage infections. EF1α-eGFP^+^ MuHV-4 was pre-incubated with mAbs or MuHV-4 immune serum (2 h, 37 °C), then added to BHK-21 (0.1 p.f.u. per cell), NMuMG (0.1 p.f.u. per cell) or RAW-264 cells (4 p.f.u. per cell). All cells were incubated overnight (37 °C) with 100 µg phosphonoacetic acid ml^−1^ and analysed for viral eGFP expression by flow cytometry. Each point shows the mean±SEM of two experiments.

SC-9E8 and SC-9A5 concentrations that potently reduced new viral eGFP expression failed to reduce cell binding by virions physically tagged with eGFP (gM-eGFP) (Fig. 3a). Indeed binding was increased, probably due to virion cross-linking generating very high avidity particles. In contrast, neutralization by immune sera or by heparin was associated with reduced cell binding. Also SC-9A5 and SC-9E8 neutralized both cell-bound and cell-free virions, whereas immune serum was substantially less effective against cell-bound virions (Fig. 3b). SC-9A5 and SC-9E8 further reduced MuHV-4 infection of IgG Fc receptor^+^ RAW-264 cells, whereas immune serum reduced BHK-21 and NMuMG cells infections (Fig. 3c) but increased RAW-264 cell infection – because FcR-dependent virion uptake bypasses the serum-mediated block to cell binding (Rosa *et al.*, 2007). Thus unlike immune serum, SC-9A5 and SC-9E8 blocked infection downstream of cell binding.

**Fig. 4.**
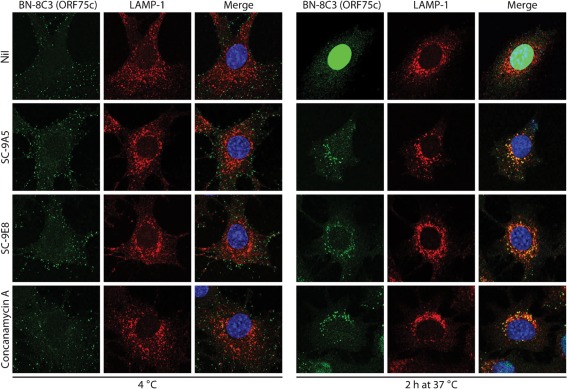
SC-9A5 and SC-9E8 prevent release of the ORF75c tegument protein. MuHV-4 (3 p.f.u. per cell) was left untreated (nil) or pre-incubated (2 h, 37 °C) with 400 µg SC-9A5 (IgG_3_) or SC-9E8 (IgG_2a_) ml^−1^ before binding to NMuMG cells (2 h, 4 °C). For concanamycin A treatment, cells were incubated with 1 µM concanamycin A before adding virus (2 h, 37 °C) and during binding (2 h, 4 °C). Unbound virions were then removed by washing and the cells either fixed immediately or after a further incubation (2 h, 37 °C) in the presence or absence of antibodies and drug. The cells were stained with the ORF75c-specific IgG_1_ BN-8C3 (green), a LAMP-1-specific mAb (red) and DAPI (blue). All images were taken with the same confocal settings. Incubating virions with the non-neutralizing gB-specific IgG_2a_ BN-1A7 or incubating cells with DMSO alone had no effect (not shown). Equivalent data were obtained in three further experiments.

We examined virus entry further by immunofluorescence (Fig. 4), using release of the abundant tegument component encoded by ORF75c (Gaspar *et al.*, 2008; Supplementary Fig. S1, available in JGV Online) as a marker of virion membrane fusion. The ORF75c of untreated virions was rapidly transported to the cell nucleus. Its increase in staining after fusion presumably reflects that not all the ORF75c in intact virions is accessible to antibody. When fusion was blocked with concanamycin A, virions were retained in late endosomes, as shown by ORF75c co-localizing with LAMP-1. SC-9A5 and SC-9E8 caused a similar retention. Therefore, they blocked membrane fusion rather than an upstream event such as virion transport to late endosomes.

### Effect of antibody on cell binding by recombinant gB

**Fig. 5.**
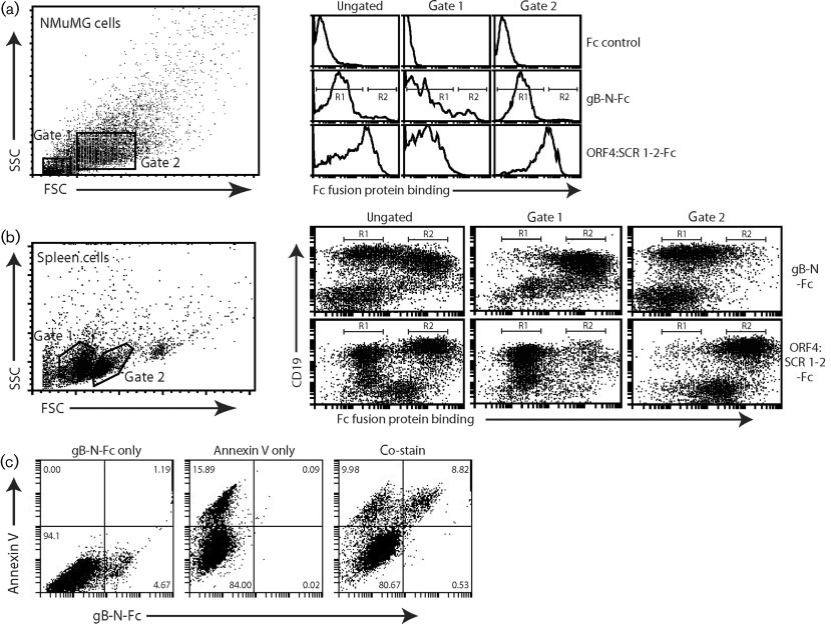
Strong staining of apoptotic cells by gB-N-Fc. (a) NMuMG cells were trypsinized, incubated overnight (37 °C) on Petri dishes, then removed by vigorous pipetting and stained with Fc fusion proteins and analysed by flow cytometry. Gate 1 corresponds to apoptotic cells (>95 % annexin V^+^) and gate 2 to viable cells (<10 % annexin V^+^). For gB-N-Fc staining of ungated cells, 2.5 % of cells were in region R2 and those in R1 had median fluorescence intensity (MFI) = 19.4; gate 1 had 7.6 % of cells in R2 with R1 MFI = 9.1; gate 2 had 0.3 % of cells in R2 with R1 MFI = 20.3. For ORF4:SCR 1-2-Fc staining, ungated cells had MFI = 88.4, gate 1 MFI = 9.8 and gate 2 MFI = 189.3. (b) The spleen of a naive mouse was disrupted into a single-cell suspension and red cells removed by centrifugation on Ficoll. The remaining cells were incubated with a blocking antibody for FcγRII/III (CD16/CD32) and then stained with Fc fusions and analysed by flow cytometry as in (a). B cells were identified by staining for CD19. Again gate 1 corresponds to apoptotic cells and gate 2 to viable cells. For gB-N-Fc staining of ungated cells, R1 contained 38.9 % of CD19^+^ cells and R2 45.1 %; gate 1 had 0.9 % of CD19^+^ in R1 and 95.8 % in R2; gate 2 had 82.9 % of CD19^+^ in R1 and 1.5 % in R2. For ORF4:SCR 1-2-Fc staining, ungated cells had 43.2 % of CD19^+^ in R1 and 49.5 % in R2; gate 1 had 95.2 % of CD19^+^ in R1 and 2.0 % in R2; gate 2 had 0.7 % of CD19^+^ in R1 and 96.6 % in R2. (c) NMuMG cells were co-stained with gB-N-Fc and with annexin V, then analysed by flow cytometry. The percentage of cells in each quadrant is shown.

We next established how SC-9A5 and SC-9E8 affected the interaction of gB with its cellular ligands. We have previously shown that gB-N fused to IgG Fc binds to cell surfaces (Gillet *et al.*, 2007b). Across a range of cell types, gB-N-Fc bound most cells weakly and a minor population strongly, suggesting two distinct modes of binding: Fig. 5(a) shows NMuMG cells and Fig. 5(b) shows spleen cells. Surprisingly, the strongly bound cell subset had lower forward and side scatter, consistent with apoptosis. IgG Fc alone did not bind to these cells, and an Fc fusion of gp70 short consensus repeats (SCRs) 1 and 2, which binds to heparan sulfate (HS) (Gillet *et al.*, 2007b), bound poorly. Therefore, the strong binding was specific to gB-N-Fc. The fact that these cells were apoptotic was confirmed by co-staining with annexin V: the cells that stained strongly by gB-N-Fc were all annexin V^+^ (Fig. 5c).

**Fig. 6.**
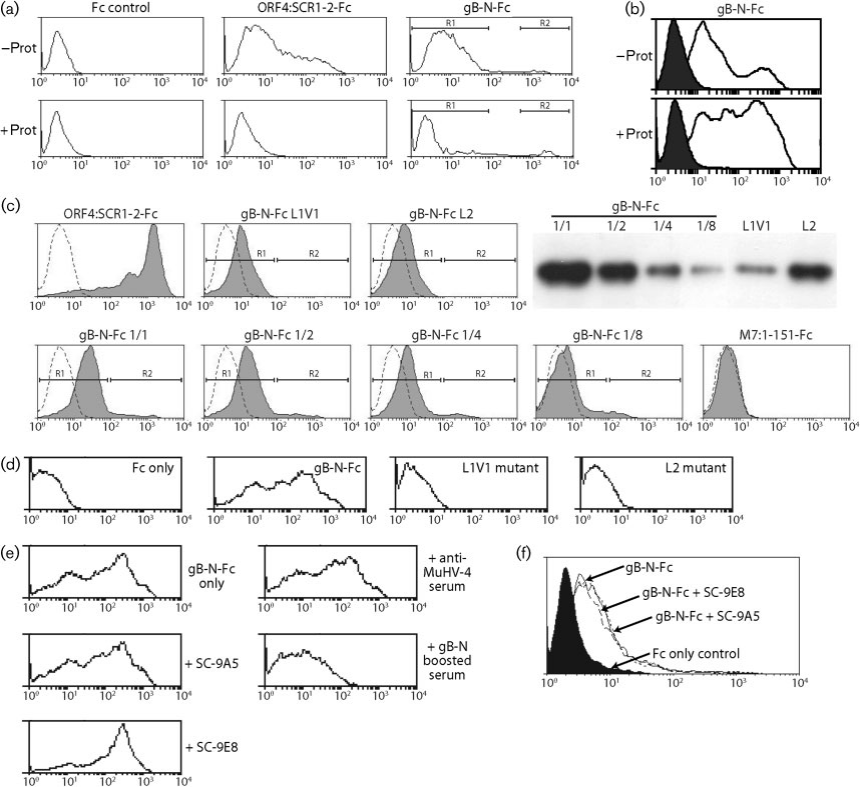
gB-N-Fc shows two distinct modes of cell binding. (a) NMuMG epithelial cells were incubated or not with proteinase K (100 µg ml^−1^, 15 min, 37 °C), then washed five times in PBS, stained with Fc fusions of the HS-binding domains of gp70 (ORF4) or gB-N as indicated, and analysed by flow cytometry. Protease treatment completely abolished ORF4:SCR1-2-Fc staining. For gB-N-Fc staining it reduced the MFI of cells in R1 from 6.2 to 1.7, while the percentage of cells in R2 increased from 1.4 to 6.3. (b) SF9 insect cells were incubated or not with proteinase K as in (a), then stained with Fc alone (filled histograms) or with gB-N-Fc (open histograms), and analysed by flow cytometry. Staining by gB-N-Fc was increased by protease treatment. (c) NMuMG cells were stained with Fc fusion proteins as indicated. ORF4:SCR1-2 binds to HS; M7 : 1-151 does not bind to NMuMG cells; L1 and L2 are fusion-loop mutants of gB-N as described in Fig. 1(e). The gB-N mutants were expressed less well than WT gB-N-Fc, so the immunoblot compares L1 and L2 supernatants with dilutions of the WT, probed for human IgG Fc. The flow cytometry profiles show NMuMG cell staining with the same supernatants (filled histogram) versus an Fc only control (open histogram). The L1 and L2 mutants lacked the minor, strongly stained population seen with all dilutions of WT gB-N-Fc, but preserved the weaker staining of all the cells. Thus, the L1 mutant had 0 % of cells in R2 with R1 MFI = 10.6, while the equivalent amount of WT (1/8 dilution) had 4.56 % of cells in R2 with R1 MFI = 5.9. The L2 mutant had 0 % of cells in R2 with R1 MFI = 8.2, while the equivalent amount of WT (1/2 dilution) had 6.4 % of cells in R2 with R1 MFI = 15.1. (d) SF9 insect cells were stained with matched concentrations of gB-N-Fc, the L1 or L2 mutants or Fc only, then analysed by flow cytometry. In contrast to NMuMG cells, the gB-N-Fc staining of SF9 cells was completely fusion loop-dependent. (e) gB-N-Fc was incubated with mAbs SC-9A5 or SC-9E8, then used to stain SF9 cells. Flow cytometry showed no loss of this binding, which in (d) was fusion loop-dependent. Nor was it blocked by serum from MuHV-4-infected mice, but serum from MuHV-4-infected mice boosted with a vaccinia virus recombinant expressing GPI-linked gB-N (boosted serum) significantly reduced gB-N-Fc binding (*P*<10^−5^ by *t*-test). (f) gB-N-Fc was incubated with mAbs SC-9A5 or SC-9E8, then used to stain NMuMG cells. Flow cytometry showed no reduction in this binding, which in (c) was fusion loop-independent.

The HSV-1 gB binds to lipids via its fusion loops (Hannah *et al.*, 2009). Protease digestion of NMuMG cells (Fig. 6a) abolished their general weak staining by gB-N-Fc, but not the strong staining of apoptotic cells, consistent with these being distinct interactions and with the latter involving a non-protein, presumably lipid ligand. In order to distinguish the two binding activities of gB-N-Fc more clearly, we used SF9 insect cells as a target likely to lack any gB protein ligand. gB-N-Fc staining of SF9 insect cells (Fig. 6b) was completely protease-resistant, implying that it was due entirely to lipid binding. That it involved the gB fusion loops was confirmed by mutating fusion loops 1 or 2 (see Fig. 1e). Mutations L1V1 and L2 abolished all staining of SF9 cells (Fig. 6d) and also the strong staining of apoptotic NMuMG cells (Fig. 6c), but not the general weak staining of NMuMG cells. Mutation L1V3 had a similar effect (data not shown). Mutation L1V2 could not be tested due to poor expression as gB-N-Fc.

mAbs SC-9A5 and SC-9E8 inhibited gB-N-Fc binding to neither SF9 (Fig. 6e) nor NMuMG cells (Fig. 6f). Therefore, their neutralization did not involve an inhibition of gB binding to either its fusion loop-independent protein ligand or its fusion loop-dependent lipid ligand.

It is important to note that MuHV-4 does not actually infect SF9 or apoptotic cells. Infection requires HS engagement (Gillet *et al.*, 2007b), and apoptotic and SF9 cells express little HS. gB binding presumably occurs only downstream in infection, as virions without HS binding also lack cell binding (Gillet *et al.*, 2009a). The fusion loop-dependent binding of gB-N-Fc to SF9 and apoptotic cells may reflect lipid similarities between their plasma membranes and the late endosomal membranes with which MuHV-4 normally fuses. For example, while late endosomes are relatively deficient in the phosphatidyl serine displayed by apoptotic cells (Balasubramanian & Schroit, 2003), they are enriched for another anionic lipid, lysobisphosphatidic acid (Kobayashi *et al.*, 1998).

### Tracking the gB conformation of SC-9A5-neutralized virions

**Fig. 7.**
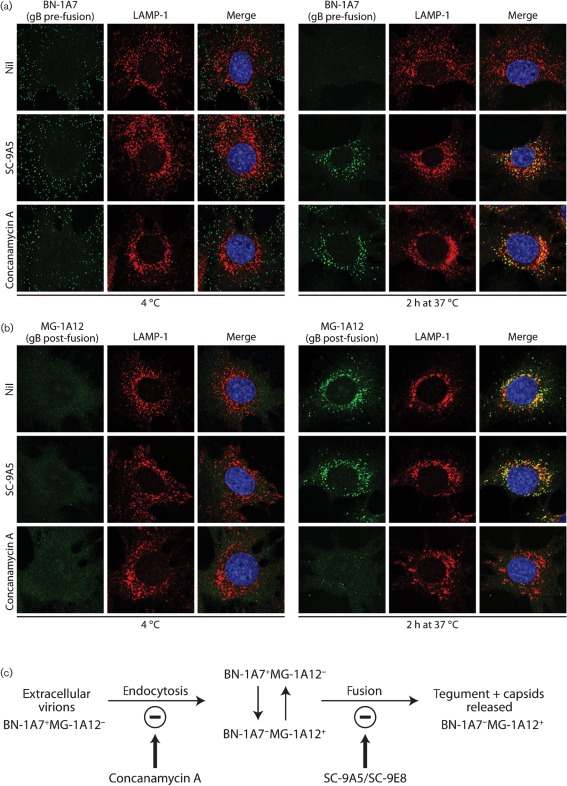
SC-9A5 arrests gB in an intermediate state between pre- and post-fusion conformations. Infections, drug treatments and antibody treatments were as for Fig. 4. In (a) the cells were stained with the pre-fusion gB-specific IgG_2a_ BN-1A7 and in (b) with the post-fusion gB-specific IgG_2a_ MG-1A12 (green). The cells were also stained for LAMP-1 (red) and with DAPI (blue). Treating cells with DMSO alone had no effect (not shown). Equivalent data were obtained in two further experiments. (c) This schematic diagram summarizes the progress of antigenic changes in gB during virion entry, and the distinct blocks to this progress effected by concanamycin A and antibody-mediated neutralization. We hypothesize that the BN-1A7^+^MG-1A12^+^ state of neutralized virions reflects their gB being able to switch reversibly between its pre- and post-fusion conformations.

MuHV-4 capsid (Gillet *et al.*, 2008a) and tegument (Fig. 4) release from late endosomes is associated with gB switching its antigenicity from BN-1A7^+^MG-1A12^−^ to BN-1A7^−^MG-1A12^+^ (Fig. 2). Virions neutralized by mAb SC-9A5 were transported to late endosomes but remained BN-1A7^+^ (Fig. 7a). To our surprise they nonetheless gained MG-1A12 reactivity (Fig. 7b). Therefore, although the post-fusion state of BN-1A7^−^MG-1A12^+^ was not achieved, consistent with ORF75c release being blocked (Fig. 4), gB did not remain in its pre-fusion form. We conclude that mAb SC-9A5 prevents membrane fusion not by blocking the initiation of gB conformation changes, but by blocking their resolution to a stable post-fusion state (Fig. 7c).

### Limitations on gB-directed neutralization

**Fig. 8.**
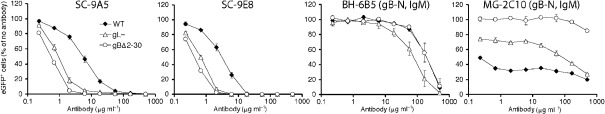
Susceptibility to gB-directed neutralization of WT, gL^−^ and gBΔ2-30 virions. WT virions or those lacking gL (gL^−^) or the N-terminal 30 residues of the mature gB (gBΔ2-30) were incubated with gB-specific mAbs as shown then added to BHK-21 cells. The cells were scored for virus infection by flow cytometric assay of viral eGFP after overnight incubation (37 °C) with 100 µg phosphonoacetic acid ml^−1^. SC-9A5 and SC-9E8 neutralized the gL^−^ and gBΔ2-30 mutants significantly better than WT (*P*<10^−5^ by chi-squared test, comparing the proportions of eGFP^+^ and eGFP^−^ cells for at least three antibody dilutions). BH-6B5 neutralized only the gL^−^ mutant better (*P*<10^−5^). The gBΔ2-30 mutant lacks the epitope recognized by mAb MG-2C10, but why the gL^−^ mutant was less well neutralized by MG-2C10 than the WT (*P*<10^−5^) was unclear. Each point shows the mean±SEM of two experiments

The MuHV-4 gB associates with gH/glycoprotein (gL) (Gillet & Stevenson, 2007b), and its N terminus hides an epitope on the gH/gL extracellular domain (Gillet & Stevenson, 2007a). This association also restricts gB-directed neutralization (Gillet *et al.*, 2009b), and gL^−^ virions were more susceptible than gL^+^ to neutralization by SC-9E8 and SC-9A5 (Fig. 8). Deleting the gB N terminus also increased gB susceptibility to these mAbs. While not compromising MuHV-4 replication, this disrupts the interaction between the gB and gH/gL extracellular domains (Gillet & Stevenson, 2007a). Therefore, despite the neutralization of wild-type (WT) virions by SC-9E8 and SC-9A5 being substantial, it was still restricted in part by the interaction between gB and gH/gL.

## Discussion

The central role of gB in herpesvirus cell binding and membrane fusion should make it a good neutralization target. However, MuHV-4 seems to resist gB-directed neutralization. gB glycosylation (Gillet & Stevenson, 2007a), the gB-gH/gL association (Gillet & Stevenson, 2007b) and post-endocytic glycoprotein conformation changes (Gillet *et al.*, 2008a) all provide protection. SC-9E8 and SC-9A5 are nevertheless the most potent MuHV-4-neutralizing mAbs identified to date, and their capacity for complete neutralization places their epitope among the most vulnerable identified for any herpesvirus gB. We aimed here to understand how this neutralization works.

Most neutralizing antibodies compete with a cellular ligand for virus binding (Knossow & Skehel, 2006). SC-9A5 and SC-9E8 interfered instead with the fusion-associated gB conformation change. This is defined by gB antigenic changes that accompany capsid and tegument release. The MuHV-4 gB conformation change seems to be substantial since mAbs recognizing multiple distinct epitopes show similar changes in recognition. The HSV-1 gB (Heldwein *et al.*, 2006) provides some analogous structural information, but its pre-fusion form and conformation change remain unidentified. Thus, the MuHV-4 gB switch from BN-1A7^+^MG-1A12^−^ pre-fusion to BN-1A7^−^MG-1A12^+^ post-fusion is very different to a local change within the post-fusion HSV-1 gB observed at low pH (Dollery *et al.*, 2010; Stampfer *et al.*, 2010).

VSV-G (Roche *et al.*, 2008) provides a more useful comparison, as both its pre- and post-fusion forms are known. For example, that mAbs against gB-N specifically recognize pre-fusion gB is consistent with the equivalent region of VSV-G being apical on the pre-fusion trimer and more caudal post-fusion (Roche *et al.*, 2007). The capacity of VSV-G to switch reversibly between its pre- and post-fusion forms independent of actual membrane fusion (Roche *et al.*, 2008) suggested that the BN-1A7^+^MG-1A12^+^ state of SC-9A5-neutralized gB might similarly reflect an equilibrium between its pre-fusion (BN-1A7^+^MG-1A12^−^) and post-fusion (BN-1A7^−^MG-1A12^+^) forms. (Somewhat less BN-1A7 staining than extracellular virions, and somewhat less MG-1A12 staining than post-fusion virions, would be hard to discern by immunofluorescence; whereas increases in staining from a background of zero were very obvious.) An intermediate form of gB simultaneously expressing the BN-1A7 and MG-1A12 epitopes seemed a less likely explanation, as the VSV-G model predicts intermediates to be both unstable and very different to either pre- or post-fusion gB. SC-9A5 did not cause a partial fusion block, since it inhibited tegument release better than concanamycin A, which kept gB completely BN-1A7^+^MG-1A12^−^. Rather it arrested entry at a qualitatively distinct point. Thus, gB may start to switch conformation reversibly before actual fusion occurs.

The MG-1A12 epitope could in theory have been revealed by displacing another virion glycoprotein. However, none of gL^−^, gp150^−^, gp70^−^, gp48^−^ and ORF28^−^ virions is constitutively MG-1A12^+^; a role for gH would seem unlikely given the redundancy of gL; and gM/gN cannot protrude far from the virion membrane (May *et al.*, 2005). We conclude that MG-1A12 epitope display reflected a change in gB itself. Thus, SC-9E8/SC-9A5 did not prevent the initiation of gB conformation changes but rather their resolution. The VSV-G conformation switch implies a transient dissociation of the pre-fusion trimer (Roche *et al.*, 2008), so SC-9A5/SC-9E8 might for example prevent the reassociation of post-fusion gB monomers.

While SC-9E8 and SC-9A5 neutralized WT virions, they were even more effective against virions lacking gL or the gB N terminus (gBΔ2-30). The difference was not in maximal neutralization, but in virion susceptibility to a given antibody dose. gBΔ2-30 and gL^−^ virions both have normal levels of gB in an apparently normal conformation (Gillet & Stevenson, 2007a; Gillet *et al.*, 2009b). However, the extracellular link between gB and gH/gL is altered. The gB N terminus is normally displaced by gH/gL dissociation in late endosomes (Gillet *et al.*, 2008b); by making this change constitutive, the gBΔ2-30 and gL^−^ mutations could increase gB exposure to antibody. That higher antibody doses were sufficient to neutralize WT virions suggested that the interaction between gH/gL and gB is dynamic. However, this needs further analysis. The key point is that the SC-9E8/SC-9A5 epitope is relatively accessible even on WT virions. Targetting it could therefore provide a general route to gammaherpesvirus neutralization.

## Methods

### 

#### Cells.

BHK-21 fibroblasts (American type culture collection CCL-10), NMuMG epithelial cells (CRL-1636), 293T cells (CRL-11268) and RAW-264 macrophages (TIB-71) were grown in Dulbecco’s modified Eagle’s medium with 2 mM glutamine, 100 U penicillin ml^−1^, 100 µg streptomycin ml^−1^ (PAA Laboratories) and 10 % FCS (complete medium) (PAA Laboratories). SF9 cells were grown in Schneider’s insect cell medium (Sigma-Aldrich), supplemented as above.

#### Plasmids.

Expression plasmids for glycosyl-phosphatidyl-inositol (GPI)-linked and human IgG_1_ Fc-linked forms of the gB extracellular domain and its N-terminal 423 aa residues (gB-N) have been described (Lopes *et al.*, 2004; Gillet *et al.*, 2007b). The GPI linkage facilitates gB expression at the cell surface; full-length recombinant gB is antigenically indistinguishable (based on staining with >50 mAbs) but is retained in the endoplasmic reticulum. We used overlap PCR to introduce mutations into the putative gB fusion loop 1 (amino acid residues 127–128 WR changed to AA = gB L1V1; residues 123–125 QLT changed to AAA = gB L1V2; residues 130–132 LTT changed to AAA = gB L1V3) and fusion loop 2 (amino acid residues 215–217 FYR changed to AAA = gB L2). These mutations were confirmed by DNA sequencing. The expression plasmids were transfected into 293T cells using Lipofectamine 2000 (Invitrogen). The cells were analysed 48 h later. IgG Fc fusion proteins were similarly harvested from transfected 293T cells. Fc fusions of gp70 short consensus repeats 1–2 (ORF4:SCR1-2-Fc), which binds to heparan sulfate (HS), and gp150 amino acid residues 1–151 (M7 : 1-151-Fc), which has no known ligand, have been described (Gillet *et al.*, 2007b). IgG Fc with a leader sequence was used as a control.

#### Viruses.

All viruses were derived from a MuHV-4 bacterial artificial chromosome (BAC) (Adler *et al.*, 2000). gL^−^ (Gillet *et al.*, 2007c) and gBΔ2-30 mutants (Gillet & Stevenson, 2007a) have been described previously. The loxP-flanked BAC cassette was removed from viral genomes by passage through NIH-3T3-CRE cells (de Lima *et al.*, 2004). For neutralization assays we used viral eGFP expression from either the HCMV IE1 promoter in the BAC cassette or an intergenic EF1α promoter (May & Stevenson, 2010). For binding assays we used MuHV-4 with eGFP-tagged gM (May *et al.*, 2008). Virus stocks were grown in BHK-21 cells (de Lima *et al.*, 2004). Cell debris was removed by low speed centrifugation (1000 ***g***, 10 min) and virions recovered from supernatants by high speed centrifugation (38 000 ***g***, 90 min). Virus stocks were titrated by plaque assay (de Lima *et al.*, 2004). After incubation with virus (2 h, 37 °C), BHK-21 cell monolayers were overlaid with 0.3 % carboxymethylcellulose (BDH) and 4 days later fixed with 4 % formaldehyde and stained with 0.1 % toluidine blue (Sigma-Aldrich).

#### Neutralization assays.

For single-cycle infections, eGFP^+^ viruses were incubated with or without antibodies (2 h, 37 °C) then added to cells (2 h, 37 °C) and cultured overnight in complete medium plus phosphonoacetic acid (100 µg ml^−1^; Sigma-Aldrich) to prevent secondary spread. The proportion of infected cells in each culture was then determined by flow cytometry of eGFP expression. For binding assays, gM-eGFP^+^ virions were incubated with or without antibodies (2 h, 37 °C) then added to cells (2 h, 4 °C) and the cells analysed directly for green fluorescence by flow cytometry.

#### Antibodies.

All MuHV-4-specific mAbs were derived from MuHV-4-infected BALB/c mice. Staining was with hybridoma supernatants. For neutralization assays, hybridoma supernatants were concentrated by ammonium sulfate precipitation, dialysed against PBS and quantified by Mancini assay (Mancini *et al.*, 1965). Immune sera were harvested >3 months post-infection and pooled from >five mice. Rat anti-mouse LAMP-1 was from BD Biosciences.

#### Drug treatments.

Concanamycin A (Sigma) stock solutions were prepared at 150 µM in DMSO. Cells were treated with 1 µM concanamycin A for 2 h at 37 °C prior to addition of virus, during virus binding at 4 °C, and during virus endocytosis at 37 °C. Treatment with identical volumes of DMSO served as a negative control for concanamycin A treatments. Porcine intestinal heparin was from Sigma.

#### Immunofluorescence.

NMuMG cells were seeded overnight on to glass coverslips. MuHV-4 virions (3 p.f.u. per cell) were bound to the cells (2 h, 4 °C). The cells were then washed three times in ice-cold PBS to remove unbound virions, and either fixed directly or first incubated (2 h, 37 °C) in complete medium with or without drugs and antibodies. After one wash in ice-cold PBS, cells were fixed by adding ice-cold 4 % formaldehyde in PBS and leaving at room temperature (RT) for 30 min (mAb BN-8C3) or 1 h (all other mAbs). The cells were then washed three times in PBS, permeabilized with 0.1 % Triton X-100 (30 min, RT), blocked (overnight, 4 °C) with 3 % BSA/0.1 % Triton X-100, then stained with primary mAbs (1 h, RT), washed three times in PBS, stained with secondary Abs diluted in 5 % normal goat serum with 1 µg DAPI ml^−1^ (1 h, RT), washed three times in PBS and once in H_2_O, and mounted in ProLong Gold (Invitrogen). Secondary antibodies (goat anti-rat IgG or goat anti-mouse IgG, IgG_1_, IgG_2a_ or IgG_3_, labelled with Alexa Fluor 488, 568) were all from Invitrogen. Images were acquired on a Leica TCS SP2 AOBS confocal laser scanning microscope with settings specific for DAPI (excitation, 405 nm; recording, 410–470 nm), Alexa Fluor 488 (excitation, 488 nm; recording, 493–560 nm) and Alexa Fluor 568 (excitation, 561 nm; recording, 566–700 nm). Images were analysed with ImageJ.

#### Flow cytometry.

Transfected or MuHV-4-infected cells were trypsinized and washed in PBS. Spleens were removed post-mortem and homogenized into single-cell suspensions. Red cells were then removed by centrifugation on Ficoll. Viral eGFP expression was visualized directly. For specific staining, cells were incubated (1 h, 4 °C) with MuHV-4 glycoprotein-specific mAbs followed by fluorescein-conjugated rabbit anti-mouse IgG pAb (Dako Cytomation) diluted in 5 % normal rabbit serum, or with IgG-Fc fusion proteins followed by fluorescein- or phycoerythrin-conjugated goat anti-human IgG pAb (Sigma) diluted in 5 % normal goat serum (1 h, 4 °C). Unlabelled anti-CD16/32 Fc blocking mAb, phycoerythrin-conjugated annexin V and fluorescein-conjugated anti-CD19 mAb were from BD Biosciences. All samples were washed twice in PBS and analysed on a FACScan or FACSCalibur (BD Biosciences).

#### Structure prediction.

A 3D-structure prediction of the MuHV-4 gB ectodomain (amino acid residues 32–730) was made using the iterative threading assembly refinement (I-TASSER) server (Zhang, 2008; Roy *et al.*, 2010). Structure models were analysed with PyMol (DeLano Scientific LLC).
